# Rivers and landscape ecology of a plant virus, Rice yellow mottle virus along the Niger Valley

**DOI:** 10.1093/ve/veab072

**Published:** 2021-08-17

**Authors:** Souley Issaka, Oumar Traoré, Régis Dimitri Skopé Longué, Agnès Pinel-Galzi, Mandev S Gill, Simon Dellicour, Paul Bastide, Stéphane Guindon, Eugénie Hébrard, Marie-Jo Dugué, Yacouba Séré, Silla Semballa, Séverin Aké, Philippe Lemey, Denis Fargette

**Affiliations:** FSA, University Boubakar Bâ of Tillabéri, Tillabéri BP 175, Niger; Laboratoire de Virologie et de Biotechnologie Végétale (LVBV), Laboratoire National de Biosécurité, Institut de l'Environnement et de Recherches Agricoles (INERA), Ouagadougou 01 BP 476, Burkina Faso; Laboratoire des Sciences Biologiques et Agronomiques pour le Développement (LaSBAD), Département des Sciences de la Vie, Université de Bangui, Bangui BP 908, République Centrafricaine; PHIM Plant Health Institute, Université de Montpellier, IRD, CIRAD, INRAE, Institut Agro., Montpellier cedex 5 BP 64501 34394, France; Department of Microbiology, Immunology and Transplantation, Rega Institute, KU Leuven, Herestraat 49, Leuven 3000, Belgium; Department of Microbiology, Immunology and Transplantation, Rega Institute, KU Leuven, Herestraat 49, Leuven 3000, Belgium; Spatial Epidemiology Lab. (SpELL), Université Libre de Bruxelles, CP160/12, 50, av. FD Roosevelt, Bruxelles 1050, Belgium; IMAG – UMR 5149, Université de Montpellier, Case courrier 051, Place Eugène Bataillon, Montpellier 34090, France; Department of Computer Science, LIRMM, CNRS and Université de Montpellier, Montpellier, France; PHIM Plant Health Institute, Université de Montpellier, IRD, CIRAD, INRAE, Institut Agro., Montpellier cedex 5 BP 64501 34394, France; Agronomy and Farming Systems, 3 avenue des Cistes, Saint Mathieu de Tréviers 34270, France; Agricultural Research and Development, Bobo-Dioulasso BP 1324, Burkina Faso; Laboratoire des Sciences Biologiques et Agronomiques pour le Développement (LaSBAD), Département des Sciences de la Vie, Université de Bangui, Bangui BP 908, République Centrafricaine; UFR Biosciences, Laboratoire de Physiologie Végétale, Université Félix Houphouët-Boigny, Abidjan 22 BP 582, Côte d’Ivoire; Department of Microbiology, Immunology and Transplantation, Rega Institute, KU Leuven, Herestraat 49, Leuven 3000, Belgium; PHIM Plant Health Institute, Université de Montpellier, IRD, CIRAD, INRAE, Institut Agro., Montpellier cedex 5 BP 64501 34394, France

**Keywords:** disease ecology, molecular epidemiology, viral phylogeography, landscape phylogeography

## Abstract

To investigate the spread of *Rice yellow mottle virus* (RYMV) along the Niger River, regular sampling of virus isolates was conducted along 500 km of the Niger Valley in the Republic of Niger and was complemented by additional sampling in neighbouring countries in West Africa and Central Africa. The spread of RYMV into and within the Republic of Niger was inferred as a continuous process using a Bayesian statistical framework applied previously to reconstruct its dispersal history in West Africa, East Africa, and Madagascar. The spatial resolution along this section of the Niger River was the highest implemented for RYMV and possibly for any plant virus. We benefited from the results of early field surveys of the disease for the validation of the phylogeographic reconstruction and from the well-documented history of rice cultivation changes along the Niger River for their interpretation. As a prerequisite, the temporal signal of the RYMV data sets was revisited in the light of recent methodological advances. The role of the hydrographic network of the Niger Basin in RYMV spread was examined, and the link between virus population dynamics and the extent of irrigated rice was assessed. RYMV was introduced along the Niger River in the Republic of Niger in the early 1980s from areas to the southwest of the country where rice was increasingly grown. Viral spread was triggered by a major irrigation scheme made of a set of rice perimeters along the river valley. The subsequent spatial and temporal host continuity and the inoculum build-up allowed for a rapid spread of RYMV along the Niger River, upstream and downstream, over hundreds of kilometres, and led to the development of severe epidemics. There was no evidence of long-distance dissemination of the virus through natural water. Floating rice in the main meanders of the Middle Niger did not contribute to virus dispersal from West Africa to Central Africa. RYMV along the Niger River is an insightful example of how agricultural intensification favours pathogen emergence and spread.

## 1. Introduction

*Rice yellow mottle virus* (RYMV) was first isolated in 1966 in East Africa (Bakker 1974) and in 1975 in West Africa (Fauquet and Thouvenel 1977). Since then, RYMV has been detected in almost all rice-producing countries of sub-Saharan Africa. RYMV is a major deterrent to rice cultivation in Africa (Abo, Sy, and Alegbejo 1997). Rice in the Republic of Niger is cultivated over 40,000 ha, mostly along the Niger River which runs over 500 km west of the country from latitudes 14° to 11° North (Issaka et al., 2012a,b). At the end of the nineteenth century, rice was already a major crop in the country (Viguier 1939). The African rice *Oryza glaberrima* was cultivated as floating rice within the meanders of the Niger River and along the shores of the valley. From the mid-1960s to the early 1990s, a major irrigation scheme extending over 8,000 ha was developed (PAFRIZ 2006). It consisted of a set of forty irrigated perimeters, several hundred hectares each, dedicated to double rice-cropping. RYMV in the Republic of Niger was reported for the first time in 1982 (Reckhaus and Adamou 1986), and severe epidemics have been described since then (John, Thottappilly, and Awoderu 1984; Awoderu 1991; Issaka et al., 2012a). Over the last decade, rice yellow mottle disease was found in almost all rice perimeters at an incidence between 5 and 100 per cent (Issaka et al., 2012a; Oludare, Tossou, and Silué 2016). Recently, a pathotype able to overcome the resistance of all known resistance genes was reported in the Republic of Niger (Hébrard et al., 2018).

RYMV is a single-stranded positive-sense RNA species of the *Sobemovirus* genus in the *Solemoviridae* family (King et al., 2018), with an approximately 4,450-nucleotide-long genome organized into five open reading frames (ORFs) (Sõmera, Sarmiento, and Truve 2015). RYMV is a stable virus and reaches a high concentration in infected plants (Fauquet and Thouvenel 1977). RYMV has a narrow host range that is restricted to the two cultivated rice species, the Asiatic rice *O. sativa* and the African rice *O. glaberrima*, wild rice species such as *O. longistaminata, O. barthii*, and *O. rufipogon*, and related wild *Poaceae* species (Bakker 1974). Perennial hosts are important virus reservoirs between rice-growing seasons. RYMV is transmitted by contact through cultural practices (Traoré et al., 2006, 2009) and by various biotic means including chrysomelid beetles (Bakker 1974), rats, and cows (Sarra and Peters 2003). There is no evidence of transmission of RYMV by seeds (Konaté, Sarra, and Traoré 2001; Allarangaye et al., 2006). However, virus dissemination through long-range transport of seeds mixed with fragments of infected leaves and straw was postulated to explain the introduction of RYMV from East Africa to West Africa at the end of the nineteenth century (Trovão et al., 2015) and to Madagascar at the end of the twentieth century (Rakotomalala et al., 2019). Transmission by contaminated water is another possible, yet undocumented, mode of transmission. The persistence of stable and highly concentrated plant viruses in natural water sources and irrigation systems is increasingly recognized (Jones 2018); this is especially true for the family *Tombusviridae* whose capsid structure is related to that of the *Sobemovirus* genus (King et al., 2018). In an experimental setting, we detected RYMV in water after percolation through soils planted with infected rice (A. Pinel-Galzi and D. Fargette, unpublished results). Moreover, rice plants cultivated along and within rivers are often uprooted and transported by the current, in particular during floods. This suggests the possibility of transmission by water as a mode of dissemination of RYMV. Altogether, we do not know all the means of transmission of RYMV or which mode or combination of modes act in a given region and at a specific period. We do not know either the distance of spread facilitated by each mode of transmission and its contribution to short-, medium-, and long-range movements.

Altogether, the current knowledge of the means of transmission of RYMV provides little information on the origin of RYMV, the dates and routes of dispersion, the dispersal dynamics, and the factors that triggered the epidemics. However, this information is critical for controlling the disease through efficient sanitary measures and durable resistance deployment. Phylogeography is an alternative approach to shed light on these key issues. The relaxed random walk (RRW) model in continuous phylogeography allows variation in dispersal velocity across branches of the phylogeny (Lemey et al., 2010). This model provides flexibility to accommodate the different means of transmission of RYMV when reconstructing the phylogenetic dispersal history of the virus. The spatiotemporal dissemination of RYMV was reconstructed under a continuous dispersal model in East Africa and West Africa (Trovão et al., 2015) and recently in the large rice-growing country of Madagascar (Rakotomalala et al., 2019). The Niger Inner Delta in Mali was the centre of virus diversification in West Africa (Pinel-Galzi et al., 2015). In the present study, virus isolates were collected in the Republic of Niger, sequenced, and compared to a large data set of isolates from West Africa and Central Africa. As a prerequisite, we revisited the temporal signal of the RYMV data sets in the light of recent methodological advances (Doizy et al., 2020; Duchêne et al., 2020). A regular sampling scheme along the 500-km-long section of the Niger River allowed us to estimate the dates and routes of the introduction of RYMV into the country. The spread along the river was reconstructed and compared to the field surveys and the changes in rice cultivation. This is our first attempt to combine field and phylogeographic studies at such a restricted spatial scale and under a specific rice agroecology (i.e. along the Niger Valley). The ecology of RYMV under different modes of rice cultivation was assessed, and the role of water transmission was examined.

## 2. Materials and methods

### 2.1 Rice cultivation along the Niger River

The Niger River is the principal river of West Africa and extends for 4,200 km. Its source is located in southeastern Guinea. The river runs in a crescent through Mali, the Republic of Niger, along northern Benin, and then through Nigeria to the Gulf of Guinea (Fig. 4A). The Niger Basin is divided into four hydrographic basins (Moris and Thom 1990), within which rice—often the African rice *O. glaberrima*—has been traditionally cultivated under a range of extensive cultivation modes (Viguier 1939; Thom and Wells 1987). (1) In the Upper Niger, from Guinea to Bamako in Mali, rice is grown as inundated rice and as flood-recession rice along the Niger River, the Bani (its main affluent), and their respective tributaries. The Upper Niger extends to the northwest of the Ivory Coast. (2) In the Niger Inner Delta up to Lake Debo, the active delta with annual floods, rice was cultivated as floating rice. Northward to Timbuktu is the lacustrine zone made of lakes and pools where rice was cultivated as flood-recession culture. (3) In the Middle Niger east of the post-deltaic region from Timbuktu to Gao (Mali), in a Sahelian climate, the hydrographic network is restricted to the Niger River within a valley a few kilometres large. Southward in the Republic of Niger is the Tillabéri to Gaya section of the Niger River, in a Soudano-Sahelian climate (14°–11° latitude north). Several tributaries on the right bank of the Niger River originate to the east of Burkina Faso. In the Middle Niger, rice was cultivated mainly as floating rice and sometimes as flood-recession rice in the largest meanders of the Niger River, with the main ones—a few hundred hectares large—at Bourem and Gao in Mali, and at Tillabéri in the Republic of Niger. The Middle Niger, which is 1,000 km long, and, more specifically, the section of the Niger Valley within the Republic of Niger, which is 500 km long, are the main focus of our study. (4) Southward is the Lower Niger that includes the basin of the Niger River and that of the Benue River, its main affluent, which extends towards Cameroon.

A wide range of irrigation schemes have been developed over the past decades along the Niger River (Moris and Thom 1990). These schemes have deeply modified and rapidly enlarged rice cultivation. In Mali, dams built on the Niger River in the 1920s and in the 1930s led to large-scale irrigated growing areas (referred to as rice perimeters), several thousands of hectares each. In the Republic of Niger, from the mid-1960s to the early 1990s, a set of canals fed by water pumping stations was built along the Niger River (PAFRIZ 2006). These canals led to forty rice perimeters, several hundred hectares each, with an elongated geometrical shape, several kilometres long and a few hundred metres large. These perimeters are clearly distinguishable on publicly available satellite images (earth.google.com). They extended over 8,000 ha. As these perimeters allowed double rice-cropping per year, a surface over 12,000 ha per year was cultivated with rice under irrigation (PAFRIZ 2006). Irrigated rice was cultivated along with floating rice, the latter being grown over approximately 30,000 ha with a declining trend over time.

### 2.2 Surveys and sampling

The forty rice perimeters spread over 500 km under a quasi-linear arrangement between Firgoun (14°53ʹN to 0°52ʹE), which is 50 km north of Tillabéri, and Gaya (11°45ʹN to 3°34ʹE) at the extreme southwest of the country (PAFRIZ 2006). A total of eighteen perimeters are located within the Tillabéri region covering 4,500 ha. In the Niamey region, seventeen perimeters covered 4,000 ha. In the Gaya region, five perimeters covered 500 ha. Along the 250-km-long valley between Tillabéri and Say, the distance between consecutive perimeters rarely exceeds a few kilometres, except between the perimeter at the south end of the Tillabéri region and that at the north end of the Niamey region, which are approximately 40 km apart. A total of forty-four isolates were collected between 1997 and 2008 (Issaka et al., 2012a) in eighteen perimeters representative of the spatial distribution of the rice perimeters (Table S1). Three isolates were also collected in the irrigated rice perimeters around the town of Diffa, to the southeast of Niger, along the Yobé River running into Lake Chad. For each sample, two to three leaves were collected on one symptomatic plant. They were considered to belong to the same viral isolate. Samples were dried at room temperature in paper envelopes and then stored at −20°C. Total RNA extractions and reverse transcription polymerase chain reaction of the coat protein (CP) gene or of the full genome were performed as described in Pinel et al., (2000).

### 2.3 ORF4 sequence data set

The sequences of the CP gene (ORF4, 720 nt) of 547 isolates from mainland Africa and Madagascar were retrieved from NCBI (1 November 2020). The ORF4 of forty-eight additional isolates from West Africa was sequenced, including twenty isolates from the Republic of Niger (Table S2). Altogether, a data set of 595 ORF4 sequences from Africa—referred to as the AF595 data set—was assembled. This data set consisted of sequences collected in twenty-four countries between 1966 and 2018 in West Africa (twelve countries), Central Africa (six countries), and East Africa (five countries), representing nearly all rice-growing countries of sub-Saharan Africa, as well as in Madagascar. The phylogeography of isolates from West Africa (Benin, Burkina Faso, Gambia, Ghana, Guinea, the Ivory Coast, Mali, Niger, Nigeria, Senegal, Sierra-Leone, and Togo) and from the west of Central Africa (Cameron, Central African Republic, and Chad) was reconstructed under a common continuous dispersal model (Trovão et al., 2015). Therefore, the 261 ORF4 sequences from West Africa and West-Central Africa—referred to as the West African data set (WA261)—were analysed together (Table S2). The ORF4 sequences of the 240 isolates from countries to the east of Central Africa (east of the Democratic Republic of the Congo, Burundi, and Rwanda) were analysed with those from East Africa (Ethiopia, Kenya, Malawi, Tanzania, and Uganda) to make the East African data set (Table S3). The ORF4 sequences of the ninety-four isolates from Madagascar make the Malagasy data set (Table S4). The data sets from East Africa and from Madagascar, which are identical to those of Rakotomalala et al. (2019), were referred to as the EA240 and Mg94 data sets, respectively. The West African, East African, and Malagasy data sets were analysed separately because of the independent epidemiological dynamics in West Africa, East Africa, and Madagascar (Trovão et al., 2015; Rakotomalala et al., 2019).

### 2.4 Full-length genome sequence

The full-length genome sequences of twenty isolates from the West African data set were retrieved from NCBI (1 November 2020). In addition, seventeen isolates, including five isolates from the Republic of the Niger, were fully sequenced (Table S2). The thirty-seven full sequences were aligned using CLUSTAL X with default parameters (Thompson, Higgins, and Gibson 1994). The pairwise homoplasy (PHI) test was applied to detect the evidence of recombination events in a set of aligned sequences (Bruen, Philippe, and Bryant 2006). This test measures the significance of the phylogenetic discrepancy across sites in an alignment and yields a *P*-value. The PHI test is implemented in SplitsTree 4 software (Bryant and Moulton 2004). The full-length sequence alignment was also screened for recombination signals using RDP version 5.5 software (Martin et al., 2020). The default settings were used for each of the seven recombination detection algorithms that RDP incorporates, with a Bonferroni corrected *P*-value cut-off of 0.001. Only recombination events detected by more than three of the seven methods implemented in RDP were considered. The neighborNet phylogenetic network of the 37 fully sequenced isolates under a HKY85 distance model was inferred (Bryant and Moulton 2004). The neighborNet phylogenetic network illustrates the genetic relationships between sequences taking into account, in the internal box-like structures, the conflicting phylogenetic signals that are possibly due to recombination events.

### 2.5 Tests of temporal signal

RYMV is a measurably evolving population; yet, its overall temporal signal is weak (Trovão et al., 2015; Rakotomalala et al., 2019) as shown by tip cluster-randomization tests in root-to-tip regressions and Bayesian inferences (Duchêne et al., 2015; Murray et al., 2016). Rather than estimating and applying three data set-specific molecular clocks (West Africa, East Africa, Madagascar), a shared molecular clock, combining the temporal signal of the three data sets, was implemented in earlier phylogeographic reconstructions (Trovão et al., 2015; Dellicour et al., 2018; Rakotomalala et al., 2019). Here, we revisited the temporal signal of the RYMV data sets (AF595, WA261, AE240, and Mg94) in the light of recent methodological advances (Doizy et al., 2020; Duchêne et al., 2020). The temporal signal of the full data set (AF595) was first visualized in PHYLOSTEMS (Doizy et al., 2020). Tip cluster-randomization tests in root-to-tip regressions and Bayesian inferences (Duchêne et al., 2015; Murray et al., 2016) were applied to each data set. The temporal signal of each data set was assessed under a Bayesian statistical framework by BETS (Duchêne et al., 2020). The time to the most recent common ancestor (TMRCA) of each data set estimated from the shared molecular clock was compared to that estimated independently from its respective data set. The TMRCAs calculated from the shared molecular clock and from the data set-specific molecular clocks, with and without the spatial model, were compared. The aim was to assess whether the temporal signal of the enlarged West African data set WA261 alone was sufficient to reconstruct the spatiotemporal spread of RYMV in West Africa.

### 2.6 Bayesian evolutionary inferences

We reconstructed time-calibrated epidemic histories using a Bayesian statistical framework implemented in BEAST version 1.10.4 software (Suchard et al., 2018). BEAST uses Markov Chain Monte Carlo integration to average over all plausible evolutionary histories for the data, as reflected by the posterior probability. All analyses were performed using the BEAGLE library to enhance computation speed (Suchard and Rambaut 2009; Ayres et al., 2012). We specified an HKY85 substitution model with a discretized gamma distribution (four categories) to model rate heterogeneity across sites. To accommodate lineage-rate variation, an uncorrelated relaxed molecular clock that models the branch rate variation according to a lognormal distribution was specified (Drummond et al., 2006). The flexible nonparametric demographic skygrid prior was selected to accommodate for variation in the rate of coalescence (Gill et al., 2013). Stationarity and mixing (e.g. based on effective sample sizes >200 for the continuous parameters) were examined using Tracer version 1.7 (Rambaut et al., 2018). The MCC trees were summarized using TreeAnnotator version 1.10.4 (Suchard et al., 2018).

To study the geographical spread of RYMV in continuous space in West Africa and to quantify its tempo and dispersal, we fitted a continuous phylogenetic diffusion model to the West African data set, modelling the changes in coordinates (latitude and longitude) along each branch in the evolutionary history as a bivariate normal random deviate (Lemey et al., 2010). As a more realistic alternative to homogeneous Brownian motion, we adopted a RRW extension that models variation in dispersal rates across branches by independently drawing branch-specific rate scalers of the RRW precision matrix from a Cauchy distribution to relax the assumption of a constant spatial diffusion rate throughout the whole tree (Lemey et al., 2010). Bayesian inference using continuous diffusion models yields a posterior distribution of the phylogenetic trees, each having ancestral nodes annotated with location estimates. The level of detail offered by the continuous phylogeographic reconstructions depends on the spatiotemporal availability of samples. For contemporaneous sampling, the early spatiotemporal spread will be represented by a limited number of ancestral phylogenetic branches. Furthermore, the lineages that have gone extinct will not be found in contemporaneous sampling. The availability of old sequences will not only help to inform the reconstructions for relatively old nodes, but they may also help to account for lineages that eventually went extinct. The availability of samples is expected to increase through time, and the patterns of spread may therefore be captured more fully in recent times. However, this does not necessarily need to imply geographically wider spread because denser sampling may also simply increase the representation of closely related sequences within the same geographical areas.

The WA261 data set was used to reconstruct the dispersal of RYMV throughout West Africa and Central Africa. A spatial jitter of 10 km was applied to the locations of the isolates. This degree of noise for identical coordinates was needed to avoid improper posteriors under the RRW model and associated inference problems (Fernandez and Steel 2000, Lemey et al., 2010). However, the 10-km jitter precluded realistic phylogeographic reconstructions at restricted spatial scales as it sometimes resulted in isolates located several kilometres away from the river. Therefore, the dispersal along the Niger River was also reconstructed from the sequences of the forty-four isolates collected along the Niger River (Table S2) applying a spatial jitter of 0.1 km to the location of each isolate. To compensate for the weak temporal signal of this data set, a temporal prior was applied to the TMRCA, with a distribution taken at the corresponding node (Ng strain) in the WA261 data set analyses. We used R functions available in the package ‘seraphim’ (Dellicour, Rose, and Pybus 2016a,b) to visualize the continuous phylogeographic reconstruction of the dispersal history of RYMV lineages into and within the Republic of Niger.

The demographic history of RYMV along the Niger River in the Republic of Niger was inferred via a skygrid coalescent tree prior and, for comparison, via an exponential growth coalescent tree prior. We employed an extension of the skygrid coalescent model that integrates external covariates in a generalized linear model (GLM) framework (Gill et al., 2016) to examine the temporal relationship between the demographic history of RYMV and the extent of irrigated rice along the section of the Niger River in the Republic of Niger.

## 3. Results

### 3.1 Temporal signal

**Figure 1.**
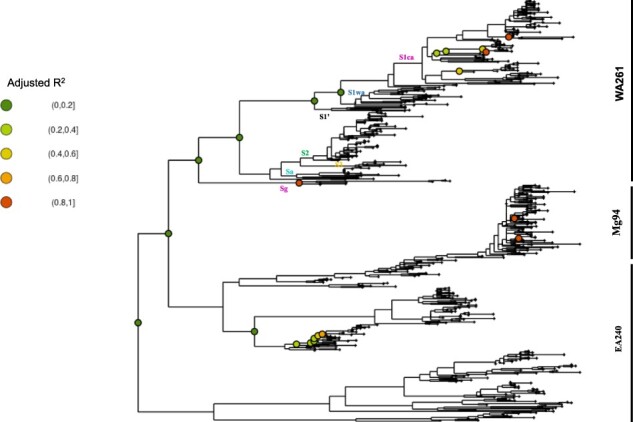
Annotated phylogenetic tree reconstructed from the AF595 data set. It underlines that the overall temporal signal was weak and heterogeneous among lineages. Coloured circles indicate nodes at which the temporal signal was found significant in PHYLOSTEMS by a root-to-tip regression analysis at a 0.01 *P*-value. The adjusted *R*^2^ for nodes with temporal signal is visualized through the colour code (upper left of the figure). The names of the strains in West Africa and West Central Africa are given at their corresponding nodes. The WA261, EA240 and Mg94 data sets are indicated along the vertical lines at the right of the figure.

We first examine the strength of the temporal signal of the data sets. Graphical representation with PHYLOSTEMS suggested that the overall temporal signal of the AF595 data set was weak (Fig. 1). The temporal signal was detected at the deepest nodes of the phylogenetic tree with a low correlation coefficient (adjusted *R*^2^ < 0.2). A temporal signal was found in a few strains only, in particular in the S1ca strain of the WA261 data set. The temporal signal was heterogeneous among the WA261, EA240, and Mg94 data sets. The temporal signal of each of the three data sets was examined by TEMPEST and BEAST using clustered tip randomization tests. The temporal signal was found in the WA261 data set both by TEMPEST and by BEAST (data not shown). In contrast, a temporal signal was detected in the EA240 data set by BEAST only and in the Mg94 data set by TEMPEST only. The analyses with BETS indicated that each of the three data sets contained a significant temporal signal with Bayes factors ranging from 43 to 107 (Table 1). The highest Bayes factor (107) was obtained with the WA261 data set, a result consistent with the clustered randomization tests. The log-relaxed clock model was better supported than the strict clock model in each of the three data sets (data not shown).

**Table 1.** Temporal signals in the data sets assessed by BETS.

Data set	Log marginal likelihood^a^		Bayes factor
	Isochronous	Dated	
West Africa			
WA261	−9627	−9520	107
East Africa			
EA240	−10,366	−10,296	70
Madagascar			
Mg94	−3703	−3660	43
Africa			
AF595	21,749	−21,636	112

a Estimated under the log-normal uncorrelated relaxed molecular clock model.

It was important to compare the TMRCA in West Africa, East Africa, and Madagascar, estimated with the shared molecular clock to that estimated from their respective data sets, as differences would impact the phylogeographic reconstructions. Interestingly, the mean TMRCA estimates and credible intervals for West Africa were similar between the shared molecular clock analyses and the individual analysis of the WA261 data set (Table 2). In addition, the estimates were robust to the incorporation of the spatial model (Table 2). For the phylogeographic reconstructions in West Africa, we therefore focused exclusively on the WA261 data set. The diffusion rate was estimated at 11 km/year (95 per cent HPD 7–15 km/year). In Madagascar too, the TMRCA estimate and the 95 per cent HPD intervals were similar whether the shared molecular clock or the Mg94-specific molecular clock was applied and whether or not the analysis included the spatial model. In contrast, the TMRCA estimates were different in East Africa—ranging from 141 to 187 years—when estimated with the shared clock or with the EA240-specific molecular clock, and whether or not including the spatial model (Table 2). Altogether, these results indicate that the temporal signal of the WA261 data set was stronger than that of the EA240 and of the Mg94 data sets. This likely results from the wider distribution of the sampling dates of the WA261 data set (Fig. S1) that covers four decades (from 1975 to 2018). The distribution of the EA240 data set (from 1996 to 2016, except the three samples of 1966) and that of the Mg94 data set (1989 to 2017) are restricted to two decades.

**Table 2.** TMRCA of clades estimated from single data sets or with the shared molecular clock^a^ with or without the spatial prior.

Data set		Shared clock		Data set-specific clock	
		–	Spatial prior	–	Spatial prior
West Africa	TMRCA	127	127	126	125
WA261	95% HPD	91–166	89–163	90–168	85–165
East Africa	TMRCA	161	141	187	169
EA240	95% HPD	104–229	93–203	128–254	111–231
Madagascar	TMRCA	36	38	37	35
Mg94	95% HPD	31–42	31–44	33–42	32–40

a The shared molecular clock was estimated from the three data sets.

### 3.2 RYMV strains in the Republic of Niger

**Figure 2.**
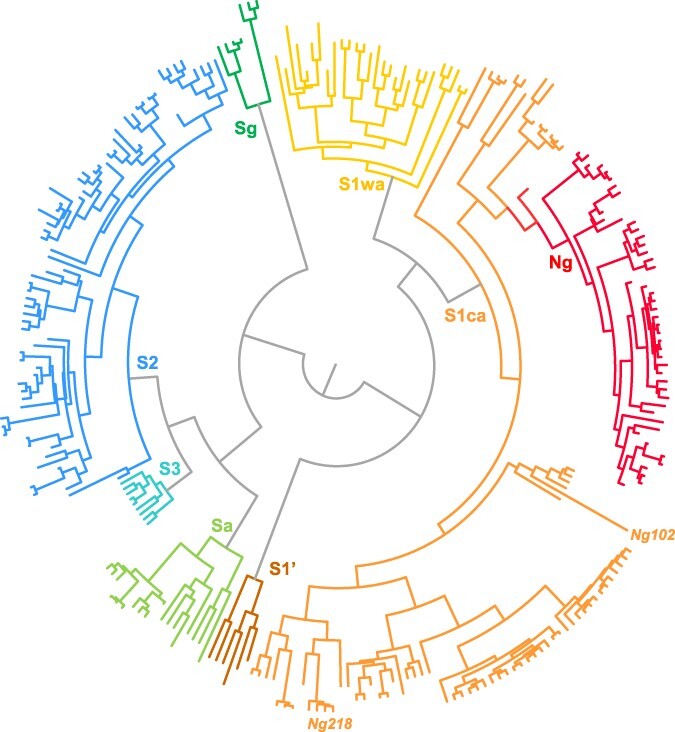
Circular phylogenetic tree of the 261 isolates of the WA261 data set reconstructed from the CP gene sequences. It displays the phylogenetic delineation of the strains.

**Figure 3.**
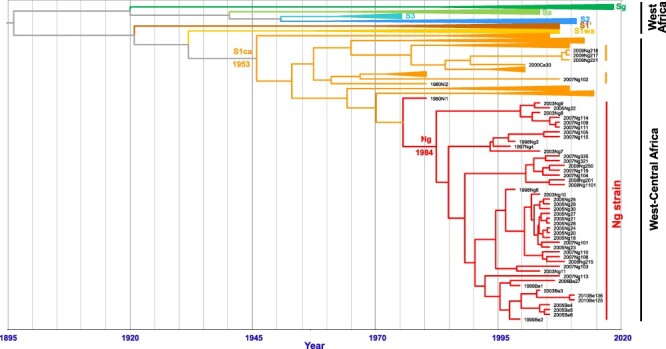
Time-calibrated phylogeny of RYMV in West Africa and in the Republic of Niger reconstructed from the CP gene sequences of 261 isolates. It shows the date of emergence of the strains. The isolates along the Niger River from the Republic of Niger and from the northeast of Benin (in red) clustered within a single strain referred to as the Ng strain. The isolates from the Diffa region near Lake Chad and isolate Ng102 are in orange. The other strains are collapsed with the name of the strain indicated. The time scale is given on the abscissa axis. The times of the most recent common ancestor of the S1ca strain and of the Ng strain are indicated.

The sequences of the 720-nt-long CP gene of 261 isolates collected between 1975 and 2018 in seventeen countries of West Africa and Central Africa were aligned. The PHI test did not find evidence of recombination (*P* = 0.90). The phylogeny reconstructed for the 261 isolates resulted in clustering patterns that were consistent with earlier phylogenetic analyses. The strains were defined on phylogenetic grounds following the nomenclature used in earlier studies (Fig. 2). The genetic diversity was strongly spatially structured, and each strain is characterized by a specific geographic distribution. A major geographical split separates isolates collected East and West of the 0.7° East longitudinal axis. All isolates collected East of this longitudinal axis belonged to a strain referred to as the West-Central African strain abridged the S1ca strain. West of this longitudinal axis are located the West-African strains (Sg, Sa, S3, S1wa, S2, and S1ʹ) (Fig. S2). The forty-one isolates from the Republic of Niger clustered within the S1ca strain. In particular, the thirty-seven isolates collected along the section of the Niger River, in the Republic of Niger, belonged to a nested strain of the S1ca strain (Figs. 2 and 3). It was hereafter referred to as the Ng strain. This strain also included seven isolates collected in Mallanville in northern Benin, on the bank of the Niger River opposite Gaya. The CP gene sequences of the forty-four isolates of the Ng strain were compiled as the Ng44 strain data set. The Ng strain showed a low genetic diversity. The isolate Ni1 sampled in the northwest of Nigeria along the Sokoto River, an affluent of the Niger River, is closely related to the Ng strain. The other isolate from the Niger Valley, isolate Ng102, was related to a strain found in northern Benin and in Nigeria. The isolates collected in the Diffa district close to Lake Chad at the East of the Republic of Niger (Ng217, Ng218, and Ng221) were related to isolates from Cameroon, Chad, and Central African Republic.

**Figure 4.**
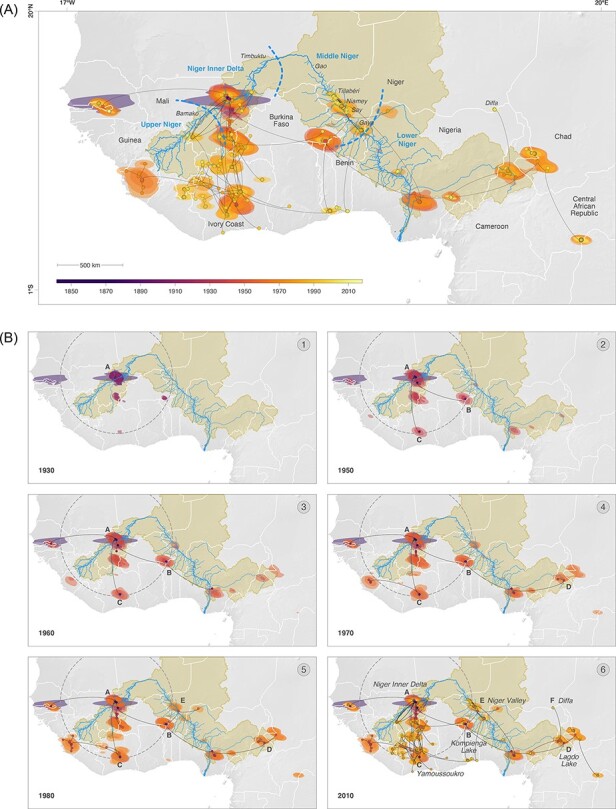
(A) Continuous phylogeographic reconstruction of the spatiotemporal dispersal of RYMV lineages in West Africa and Central Africa. For each clade, we mapped the maximum clade credibility (MCC) tree and the 95 per cent highest posterior density (HPD) regions reflecting the uncertainty related to the phylogeographic inference. MCC tree nodes and 95 per cent HPD regions are based on 1,000 trees subsampled from each post burn-in posterior distribution and coloured according to their time of occurrence. We computed such 95 per cent HPD regions for successive time slices encompassing 10 years. For a given time slice, the 95 per cent HPD region was computed by considering the position of all nodes (i.e. across all 1,000 posterior trees) occurring during this specific time slice. (B). Visualization of the continuous phylogeographic reconstruction of RYMV lineages in West Africa and Central Africa from 1930 to 2010 at intervals that capture the major dispersal events. The grey dotted circle is located 800 km away from the Niger Inner Delta (A). The Niger Inner Delta is the diversification centre in West Africa (see text). The Kompienga Lake in Burkina Faso (B), Yamoussoukro in Ivory Coast (C), the Lagdo Lake in Cameroon (D), the Niger Valley (E), and the Diffa district (F) in the Republic of Niger are indicated.

A total of thirty-seven isolates from West Africa and West-Central Africa, including eight isolates from the Republic of Niger, were fully sequenced and aligned. The PHI test found significant evidence (*P* < 10^–3^) for recombination in the WA37 full-length genome data set. However, none of the isolates from the Republic of Niger was identified as a putative recombinant through RDP analyses. The genetic and geographic relationships between the isolates of the Republic of Niger and the isolates of the other strains were assessed from the full gene sequences through a neighborNet phylogenetic network (Fig. S2). The relationships were consistent with those inferred from the CP gene (Fig. 2).

### 3.3 Introduction of RYMV west and east of the Republic of Niger

The introduction of RYMV in the Niger Valley to the west of the country and in the Diffa district to the east of the country, close to Lake Chad, had different geographic and genetic origins (Fig. 4A). They both originated from areas where rice cultivation had been extended after the construction of dams in the early 1980s. RYMV was introduced in the Diffa rice perimeters from the Lake Lagdo region in Cameroon (Fig. 4F). The Lagdo Lake (8°53ʹN; 13°58ʹE) is a 600-km^2^-large lake north of Cameroon built along the Benue River in the Lower Niger; it allows over 600 ha of rice cultivation. RYMV in the Niger Valley was introduced from the Kompienga Lake area (11°04ʹN; 0°42ʹE) in Burkina Faso southwest of the Republic of Niger (Fig. 4E). The Kompienga Lake is a 200-km^2^-large lake built along the Kompienga River that allows irrigated rice cultivation. Interestingly, RYMV also spread southward from both areas (Fig. 4F). Isolate Ng102, which belonged to a strain found in southern Nigeria, showed that RYMV was introduced more than once into the Niger Valley. The TMRCA of RYMV in the Niger Valley (i.e. that of the Ng strain) was ca. 1984 (95 per cent HPD: 1977–1991) (Fig. 3). This is relatively recent considering that the virus had circulated in West-Central Africa decades before (i.e. 1953; 95 per cent HPD: 1941–1962) (Fig. 3) and that rice had been cultivated along the Niger River centuries earlier.

### 3.4 Introduction of RYMV south of Ivory Coast

To investigate the cause of the recent introduction of RYMV in the Niger Valley, we compared it to the date of introduction of the virus in the south of Ivory Coast. The southern Ivory Coast is located at the same distance from the Niger Inner Delta as the section of the Niger Valley in the Republic of Niger (ca. 800 km). The Niger Inner Delta in Mali was the centre of virus diversification in West Africa (Pinel-Galzi et al., 2015). RYMV was introduced much earlier in Ivory Coast. The phylogeographic reconstructions showed that RYMV was present in the early 1950s in southern Ivory Coast (Fig. 4B). The wave-front rate in West Africa was 25 km/year (95 per cent HPD 16–36 km/year). Therefore, the spread from the Niger Inner Delta to the southern Ivory Coast—800 km in approximately 20 years, i.e. 40 km/year—was close to the upper range of the wave-front rate distribution. In contrast, that to the section of Niger River in the Republic of Niger—800 km in 50 years, i.e. 16 km/year—was at the lower range of the distribution. RYMV was already present in the early 1950s around Kompienga Lake (Fig. 4B), which is located at a similar distance from the Niger Inner Delta. Kompienga Lake is only 200 km away from the Niger Valley. There are no physical or agro-climatic obstacles to RYMV movement between the two regions to account for the delayed introduction. Moreover, several tributaries of the Niger River originated east of Burkina Faso. We thus explored an alternative hypothesis, i.e. whether a major and recent change in rice cultivation triggered the introduction of RYMV in this section of the Niger Valley.

The estimated date of introduction of RYMV in the Niger Valley (1984) broadly coincides with the year of the first field reports (1982). In contrast, in other countries of West Africa, the disease was often observed decades after the introduction of the virus (Fig. S3), frequently in irrigated perimeters. In the southern Ivory Coast, RYMV was first observed in 1975 (Fauquet and Thouvenel 1977). Orange-yellow patches of infected plants were noticed in several irrigated perimeters around the towns of Yamoussoukro, Gagnoa, and Lam-To. Interestingly, the disease remained unnoticed in rice fields despite frequent and detailed disease surveys conducted in the 1960s in the Ivory Coast (Davet 1962; Ravisé 1962; Boisson 1964). This implies that the first field report of infected plants is an indicator of the epidemiological stage of the disease rather than of the introduction of the virus, which remained unnoticed, either because the disease was at a low incidence in scattered rice fields or because the virus mainly circulated in wild graminaceous plants at that time. Accordingly, the correspondence between the date of introduction of the virus and the first disease report in the section of the Niger River under study reflected that rice cultivation at that time became prone to epidemic development.

### 3.5 Dissemination along the Niger River

**Figure 5.**
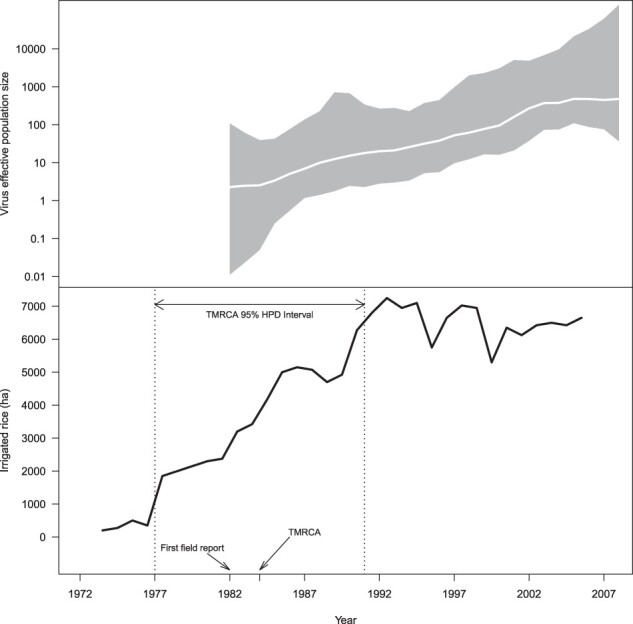
Demographic history of RYMV and surfaces of irrigated rice. Top: the demographic history of RYMV along the Niger River in the Republic of Niger was inferred via a skygrid coalescent tree prior. The intervals (in grey) represent 95 per cent HPD of the product of generation time and effective population size *N*_e_(*t*). The middle line (in white) tracks the inferred median of *N*_e_(*t*). Bottom: the surfaces (ha) of cultivated rice in the irrigated perimeters along the Niger Valley in the Republic of Niger from 1973 to 2005 (for one crop cycle) are indicated by the black curve. Arrows indicate the first field report and the TMRCA of the Ng strain. The 95 per cent HPD interval of the TMRCA is shown at the top of the figure.

**Figure 6.**
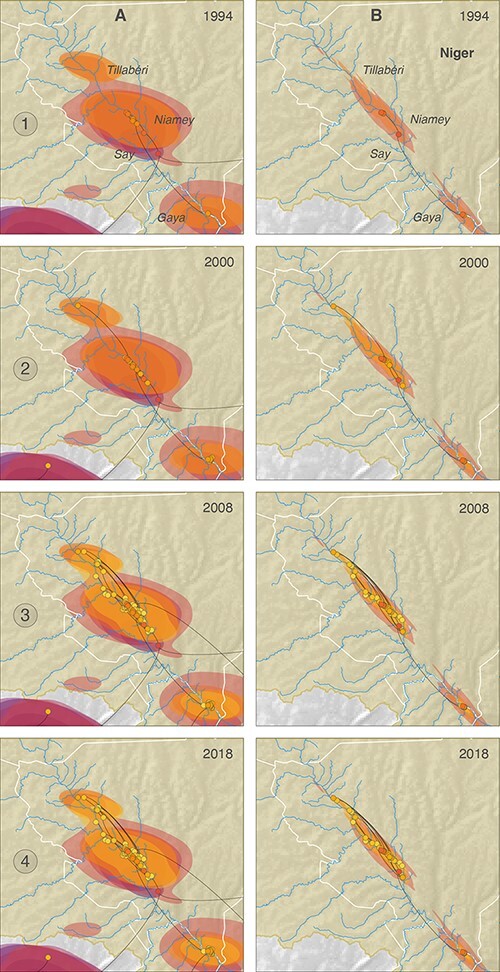
Movements of RYMV between rice perimeters along the Niger River reconstructed from the phylogeographic relationships between the forty-four isolates collected in seventeen rice perimeters reconstructed from the WA261 data set (column A) and from the Ng44 data set (column B). The coloured clouds represent statistical uncertainty in the estimated locations of internal nodes (95 per cent HPD).

Only a few hundred hectares were cultivated under irrigation before 1977. The first major development occurred in 1977 and reached approximately 2,000 ha in 1980 (Fig. 5). Since then, the surfaces rapidly increased to ca. 7,000 ha in the early 1990s. The 95 per cent HPD interval of the TMRCA of the Ng strain is 1977–1991. This interval can be narrowed to 1977–1982 when taking into account the first report of RYMV in the Republic of Niger in 1982. Then, the introduction of RYMV in the Republic of Niger matched the first stage of the development of irrigated rice cultivation in the country (Fig. 5), at a time where the vast majority of rice cultivation was still grown as floating rice (over 10,000 ha). The demographic reconstruction under a Bayesian skygrid reconstruction (Fig. 5) and under an exponential model (Fig. S4) depicted similar demographic histories, i.e. an exponential increase in effective population size after the introduction of RYMV along the Niger Valley. This rapid increase in virus population paralleled the increase in irrigated surfaces from the late 1970s to the early 1990s (Fig. 5). Phylogeographic reconstructions of the spread along the Niger Valley from the WA261 data set (Fig. 6A) and from the Ng44 data set were consistent (Fig. 6B). The Ng strain was introduced from the southern region of the country to the rice perimeters close to Say, 55 km southward of Niamey (Fig. 6A). RYMV subsequently spread rapidly to the rice perimeters of the Niamey region 50 km northward and to those of Gaya 230 km southward (Fig. 6B). RYMV later spread to the perimeters of the Tillabéri region, which are 100 km north of Niamey (Fig. 6C). This was followed by frequent movements between the Niamey and Tillabéri rice perimeters in either direction (Fig. 6D). Altogether, after its introduction along the Niger River, RYMV rapidly spread both upstream and downstream along the Niger Valley. There was no evidence of preferential downstream spread as expected under water transmission.

### 3.6 Relationship between RYMV population dynamics and rice cultivation

We examined the relationship between RYMV and rice cultivation through a skygrid–GLM coalescent model. In this model, the relationship between the viral population dynamics and a time-varying covariate is characterized by an effect size coefficient in a log-linear regression model. In this study, the covariate of interest was the time-varying surface area (ha) of cultivated rice in the irrigated perimeters along the Niger Valley in the Republic of Niger. Importantly, the skygrid–GLM jointly infers the RYMV effective population size along with the effect size coefficient that relates it to the covariate. This stands in contrast to post hoc approaches that ignore demographic uncertainty by comparing point estimates of the effective population size and covariate values (using, e.g., standard approaches for time-series comparisons). Thanks to this joint inference, the effect size coefficient in our analysis accounts for the uncertainty in the demographic reconstruction. The effect size has a posterior mean of 0.002, with a 95 per cent Bayesian credibility interval (BCI) of [−0.0006, 0.0049] (Fig. S5). Because we used the log transform of the effective population size in our model, an effect size of 0.002 is interpreted as follows: during a given year, there is a mean 0.2-per cent increase of the viral population size associated with an increase of one cultivated hectare over the number of hectares cultivated during the previous year. However, while the vast majority (>94 per cent) of the mass of the effect size posterior distribution is positive, showing some support for this association, the 95 per cent BCI includes zero and we conclude that the hypothesized relationship between the viral population size and cultivated hectares is not statistically significant.

As depicted in Fig. 5, the effective population size trajectory is monotonic (primarily increasing), while the covariate exhibits dips in the late 1980s, as well as in 1995 and 1999 (Fig. 5). The dips in 1995 and 1999 reflect major floods that occurred late in the rice-growing seasons and devastated a substantial proportion of the rice fields (Mahé et al., 2011). The rice surfaces were recorded at harvesting time (accounting for the dips in the covariate), but prior to the floods, the rice fields were planted and developed as usual and may have had a larger contribution to the RYMV dynamics than the covariate values suggested. We conducted follow-up skygrid–GLM analyses by smoothing the dips in the covariate in 1995 and 1999 by replacing the covariate values for those years with 3-year and 5-year centred averages. In both of these analyses, the inferred effect sizes still have 95 per cent BCIs that include zero.

Furthermore, we performed a skygrid–GLM analysis with more comprehensive smoothing of covariate fluctuations by replacing all covariate values with 3-year centred averages. Again, the inferred effect size falls short of statistical significance. Thus, the lack of a statistically significant relationship between the viral population size and rice surface area is not due to the major dips in the covariate in the 1990s or other fluctuations. Rather, it is consistent with a long-term divergence between dynamics of the viral population and the covariate: while the effective population size displays an increasing trend for most of the population history, the covariate increases until it peaks in 1992, after which it exhibits a long period of decrease with values that are strictly less than its peak value.

## 4. Discussion

A strong temporal and spatial signal is required to reconstruct the spatiotemporal spread of RYMV along the Niger River and to compare it to early field reports and to recent changes in rice cultivation. However, the temporal signal in earlier data sets of heterochronous sequences of RYMV was shown to be weak (Trovão et al., 2015). Previous phylogeographic studies have therefore combined data sets from West Africa, East Africa, and Madagascar to maximize the temporal signal by estimating a shared evolutionary rate (Trovão et al., 2015; Rakotomalala et al., 2019). These studies assumed that the evolutionary rates in West Africa, East Africa, and Madagascar are similar. This questionable assumption is no longer required in West Africa. The temporal signal of an enlarged data set of isolates from West Africa collected over four decades, as assessed under a Bayesian statistical framework, was sufficient to obtain reliable TMRCA estimates. These results indicated that, in this section of the Niger River, phylogeographic studies can be applied at spatial and temporal scales similar to those usually implemented in field surveys. Then, the different sources of information were combined to study the relationships between agricultural changes, virus spread, and plant disease epidemics.

The phylogeography of RYMV in the Republic of the Niger displayed the following features. The isolates collected in the Republic of Niger belonged to the West-Central African lineage. The geographical split with the West African strain matched the geographical isolation between a region of continuous rice cultivation to the west of West Africa and a region with discontinuous rice cultivation at the east (Portères 1950). This isolation lasted until rice cultivation was intensified in recent decades (Pinel-Galzi et al., 2015). The introductions of RYMV to the west and east of Niger were recent and had different geographic and genetic origins. They arose from strains circulating a few decades earlier to the south-west and south-east of the country, respectively. These introductions both originated in areas where dams had been built in the early 1980s allowing the cultivation of irrigated rice. Strain circulation around large natural lakes such as Victoria Lake and Tanganyika Lake was reported in East Africa (Ochola et al., 2015). Our study suggests that irrigated rice around smaller and recent artificial lakes in West Africa also played an active role in RYMV spread. RYMV along the Niger River was introduced from the southwest of the country. Phylogeographic reconstructions suggested that RYMV originated ca. 1984 in the rice perimeters of Say, which is among the oldest and largest rice perimeters of the country. Interestingly, the Diamangou, Goroubi, and Tapoa Rivers—where flood-recession rice is cultivated—come from the east of Burkina Faso and join the Niger River close to Say, offering corridors of dissemination from irrigated rice surfaces around the Kompienga and Bagré Lakes (Nébié 1993). After its introduction, RYMV rapidly spread northward to the rice perimeters of the Niamey and Tillabéri regions and southward to those of Gaya. Within less than two decades, RYMV spread across 500 km along the Niger River. As RYMV has rapidly spread upstream of the Niger River, there is no evidence of downstream transmission by natural water, as documented for other stable and highly concentrated plant viruses (Jones 2018). However, this does not preclude local water transmission in irrigation systems where virus concentration is likely to be much higher.

Reckhaus and Adamou (1986) stated: ‘In the Niger, virus-like symptoms were observed for the first time during the 1982 rainy season … The first cases of the disease in Niger were observed in the rice scheme of Say … During the following growing seasons, RYMV spread into other rice schemes … The disease finally became established in almost all rice-producing areas’. The observations of the field surveys and the main inferences from the phylogeographic reconstructions are consistent despite being based on different sources of information. Similar results obtained by the two methods strengthen our conclusions on the process of dispersal. Correspondence between field and phylogeographic studies at a wider spatial scale was already noticed in Madagascar (Rakotomalala et al., 2019). Interestingly, the field surveys of RYMV in Madagascar and in the Republic of Niger involved the same scientist, P. M. Reckhaus (Reckhaus and Adamou 1986; Reckhaus and Randrianangaly 1990; Reckhaus and Andriamasintseheno 1997). Differences in the disease identification procedure can therefore be excluded. In Madagascar, the rice landscape ecology was dense and continuous enough to allow rapid spread soon after virus introduction (Rakotomalala et al., 2019).

Consistently, the introduction of RYMV in the Niger Valley coincided with the creation of irrigated rice perimeters along the Niger River since the 1970s. These perimeters led to a sharp increase in the surface area of cultivated rice, which grew from a few hundred hectares in 1975 to 2,000 ha in 1977. Similarly, in the Diffa district to the east of Niger, RYMV was detected after the settlement of irrigated perimeters. The irrigated perimeters along the Niger River deeply affected the ecology of the disease. Double rice-cropping and asynchronous field work where rice nurseries coexist with ready-to-harvest fields favoured the maintenance of the virus between seasons. Perennial graminaceous are left along the irrigation canals to sustain soil stability. They are often infected (Issaka et al., 2012a) and contribute to virus survival and spread. The quasi-continuous linear layout of the rice perimeters along this section of the Niger Valley resulted in a narrow strip of rice land over 200 kilometres long. Such a rice corridor facilitates beetle transmission of the virus among rice perimeters. It also ensured human-mediated dissemination of RYMV by easing the transport of plantlets from infected seedbeds to rice perimeters (Traoré et al., 2006). Dense planting of susceptible *O. sativa* cultivars (CNEVV 2012) led to rapid inoculum build-up (Traoré et al., 2009). All these conditions sustain virus survival, virus spread, and disease epidemics and account for the link between RYMV spread and irrigated rice. In contrast, floating rice is not favourable to virus dissemination: the *O. glaberrima* varieties used are often resistant (S. Issaka, personal observations), the wild grasses, a potent reservoir of RYMV, are eliminated by the flooding, and the meanders of the river are located dozens of kilometres apart. These conditions do not allow virus spread or even virus survival. Consequently, any virus introduction in this section of the Niger River, prior to the settlement of irrigated perimeters, likely remained localized or became extinct. Consistently, floating rice was ranked as the mode of rice cultivation with the lowest disease incidence in West Africa (Awoderu 1991), and RYMV was not observed in floating rice in surveys of the Gao and Tillabéri meanders (O. Traoré and S. Issaka, personal observations). Cultivation of floating rice in distant meanders between Timbuktu and Tillabéri did not make this section of the Niger Valley a powerful enough source for virus circulation. Accordingly, RYMV was not introduced from the upstream section of the Niger River to the North (15° latitude) in Mali, as expected if the virus had spread from the Niger Inner Delta downstream the Niger Valley.

The results of the skygrid–GLM analyses showed some support for a positive association between RYMV population dynamics and irrigated rice surfaces; however, the relationship fell short of being statistically significant. There are several possible explanations for this result. First, beyond a certain surface area, the rice surface area may not be a limiting factor for virus dynamics. Second, rice seedbeds are a major virus source for rice fields (Traoré et al., 2006). Cultural practices have changed over the years: initially, the rice seedbeds were synchronous, but they recently became asynchronous and constitute a powerful source of the inoculum throughout the rice-growing season. This recent change in practice could account for the persistent viral population increase, even after the rice surfaces stabilized. Finally, the perennial wild rice *O. longistaminata* and other graminaceous species are frequent and often infected within and along rice fields (Issaka et al., 2012a). In such a case, viral dynamics will have occurred in both the cultivated and wild rice species, accounting for the disconnection between the virus population size and the rice surfaces. In any case, our skygrid–GLM analyses illustrate the difficulty in establishing a clear statistical link between RYMV dynamics and rice density, despite all of the information available on RYMV ecology that suggests a close link between RYMV epidemiology and rice. Such difficulties have been encountered in other studies of RYMV dynamics by Dellicour et al., (2018) and Rakotomalala et al., (2019). The relationship between RYMV epidemiology and rice is likely more complex than what has been posited in these modelling attempts, and future investigations that account for a combination of several factors may be illuminating.

Our study that focuses on detailed genetic, pathogenic, and historical information over narrow temporal and spatial scales reveals the role of the Niger Valley and rice intensification in virus spread. Prior to the irrigation schemes, the Middle Niger, where rice has been cultivated as floating rice, was not a corridor of propagation. Therefore, it did not account for the rapid dispersal of RYMV from West Africa to West-Central Africa. Other ways and, possibly, other means of dissemination were involved. After irrigation, sections of the Middle Niger became efficient corridors of propagation. This could contribute to the dispersion of the virulent strain recently identified in this area (Hébrard et al., 2018). The contrasting epidemiology of RYMV in floating rice and in irrigated rice shows how agricultural intensification favours pathogen dissemination. Similarly in Nigeria, RYMV was reported along the Niger and Benue Rivers in the early 1980s after the shift from traditional rice cultivation towards continuous cropping under irrigation (Rossel, Thottappilly, and Buddenhagen 1982). However, prior to rice irrigation, the epidemiology in different sections of the Niger Valley was already contrasted. For instance, the early introduction of RYMV in southern Ivory Coast in the first half of the twentieth century reflected an agro-ecosystem favourable to virus spread in the Upper Niger (Viguier 1939) and to the west of Ivory Coast (Miège 1954). Altogether, RYMV along the Niger River is an insightful example of how agricultural intensification favours pathogen emergence and spread.

## Supplementary Material

veab072_Supp
