# Supplementary material for: Rivers and landscape ecology of a plant virus, Rice yellow mottle virus along the Niger Valley
Source: Virus Evol. 2021 Aug 17;7(2):veab072. doi: 10.1093/ve/veab072 (PMC9927878; doi:10.1093/ve/veab072)
Supplement: veab072_Supp [file veab072_supp.zip › Supplementary files for review.pdf]

Figure S1

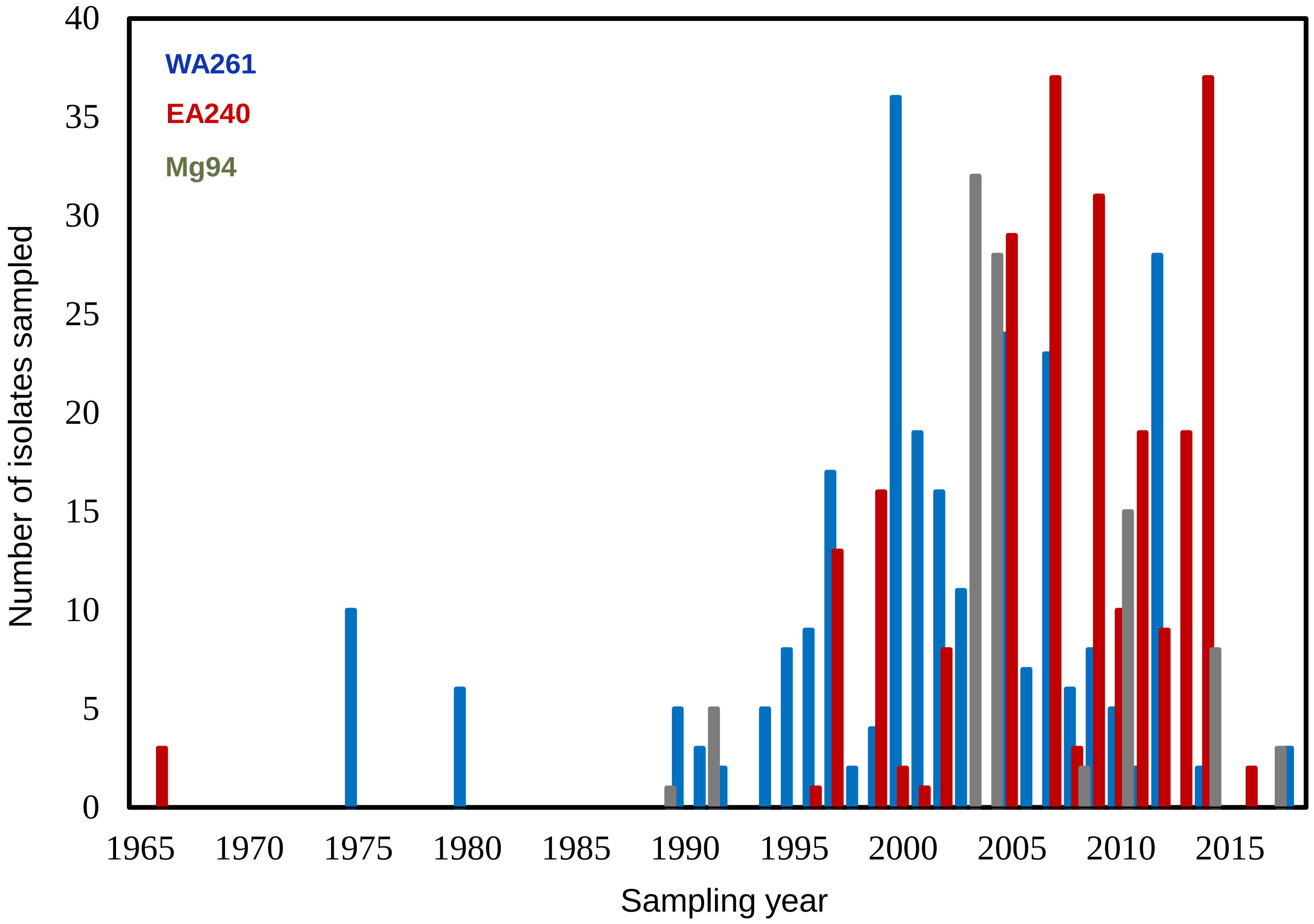

WA261
EA240
Mg94
Number of isolates sampled
0
5
10
15
20
25
30
35
40
1965
1970
1975
1980
1985
1990
1995
2000
2005
2010
2015
Sampling year

Figure S2

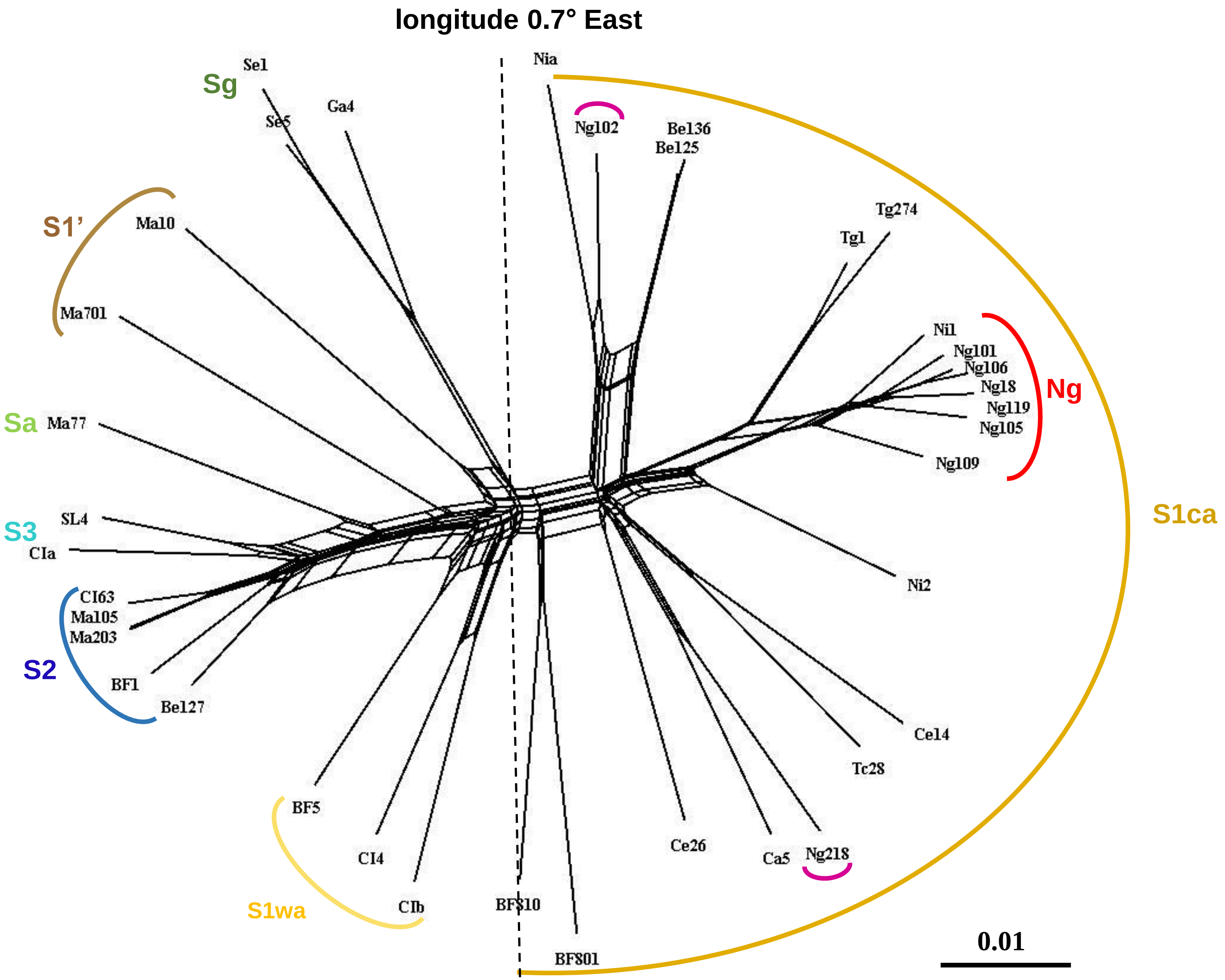
longitude 0.7° East
Se1
Sg
Se5
Ga4
Nia
Ngl02
Bel36
Bel25
S1'
Mal0
Ma701
Tg274
Tgl
Nil
Ngl01
Ngl06
Ngl8
Ng
Ngl19
Ngl05
Ngl09
Sa
Ma77
SL4
S3
CIa
S1ca
Ni2
CI63
Mal05
Ma203
S2
BF1
Bel27
Cel4
Tc28
BF5
CI4
Ce26
Ca5
Ng218
S1wa
CIb
BF810
BF801
0.01

Figure S3

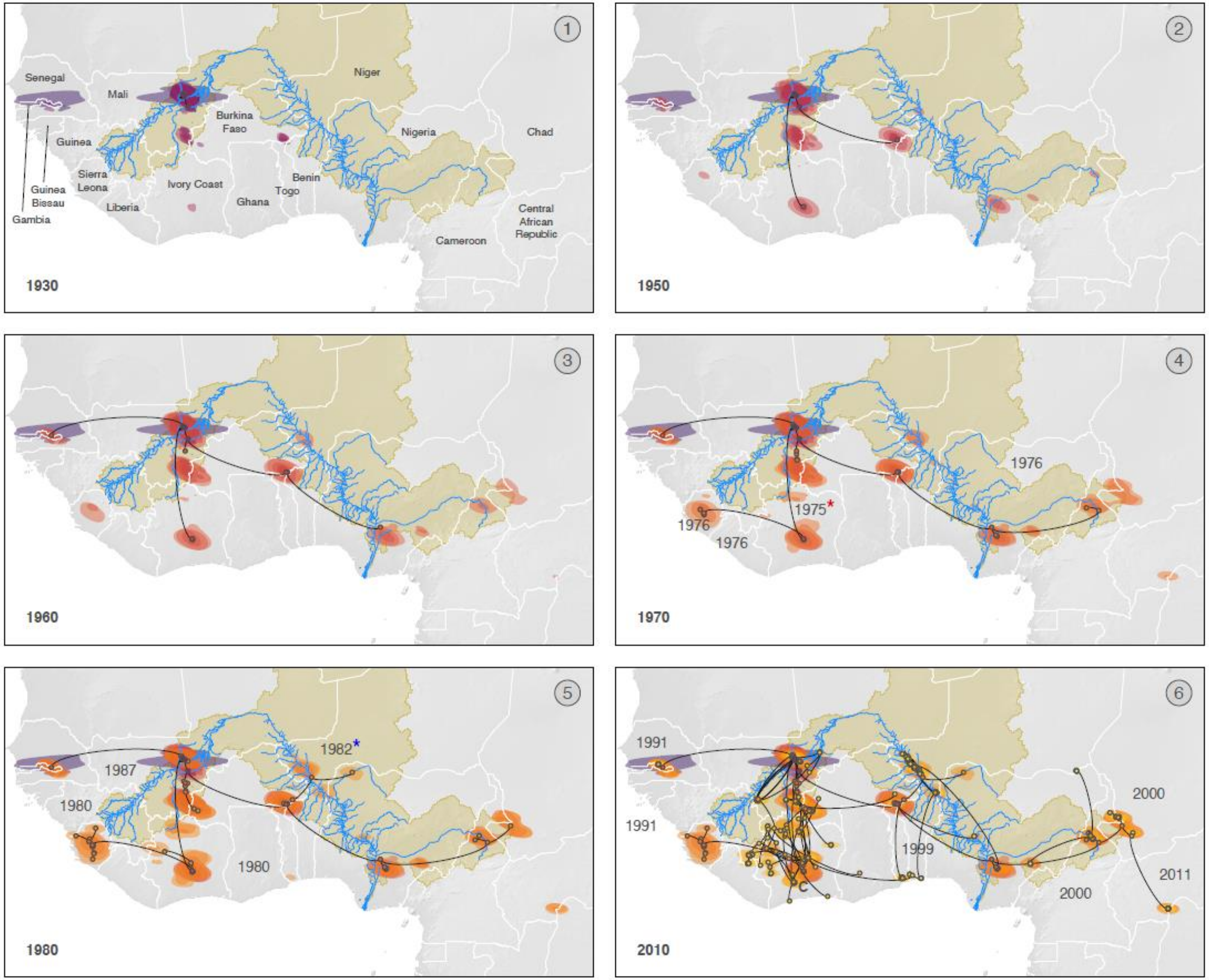
1
Senegal
Mali
Niger
Burkina Faso
Guinea
Nigeria
Chad
Sierra Leone
Guinea Bissau
Gambia
Ivory Coast
Liberia
Ghana
Benin
Togo
Central African Republic
Cameroon
1930
2
1950
3
1960
4
1976
1975*
1976
1976
1970
5
1982*
1987
1980
1980
1980
6
1991
1991
2000
1999
2000
2011
2010

Figure S4

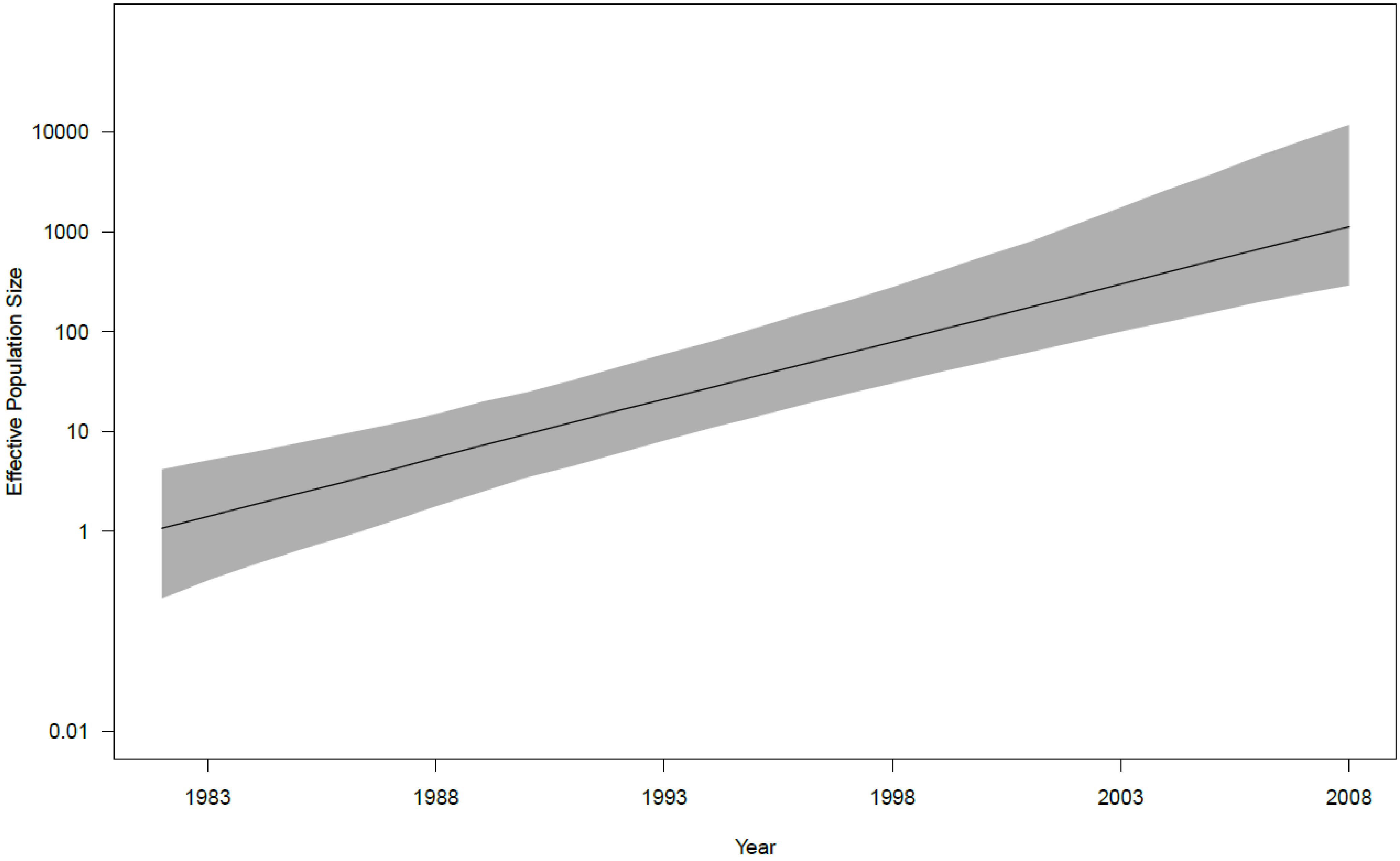

Effective Population Size
10000
1000
100
10
1
0.01
1983
1988
1993
1998
2003
2008
Year

Figure S5

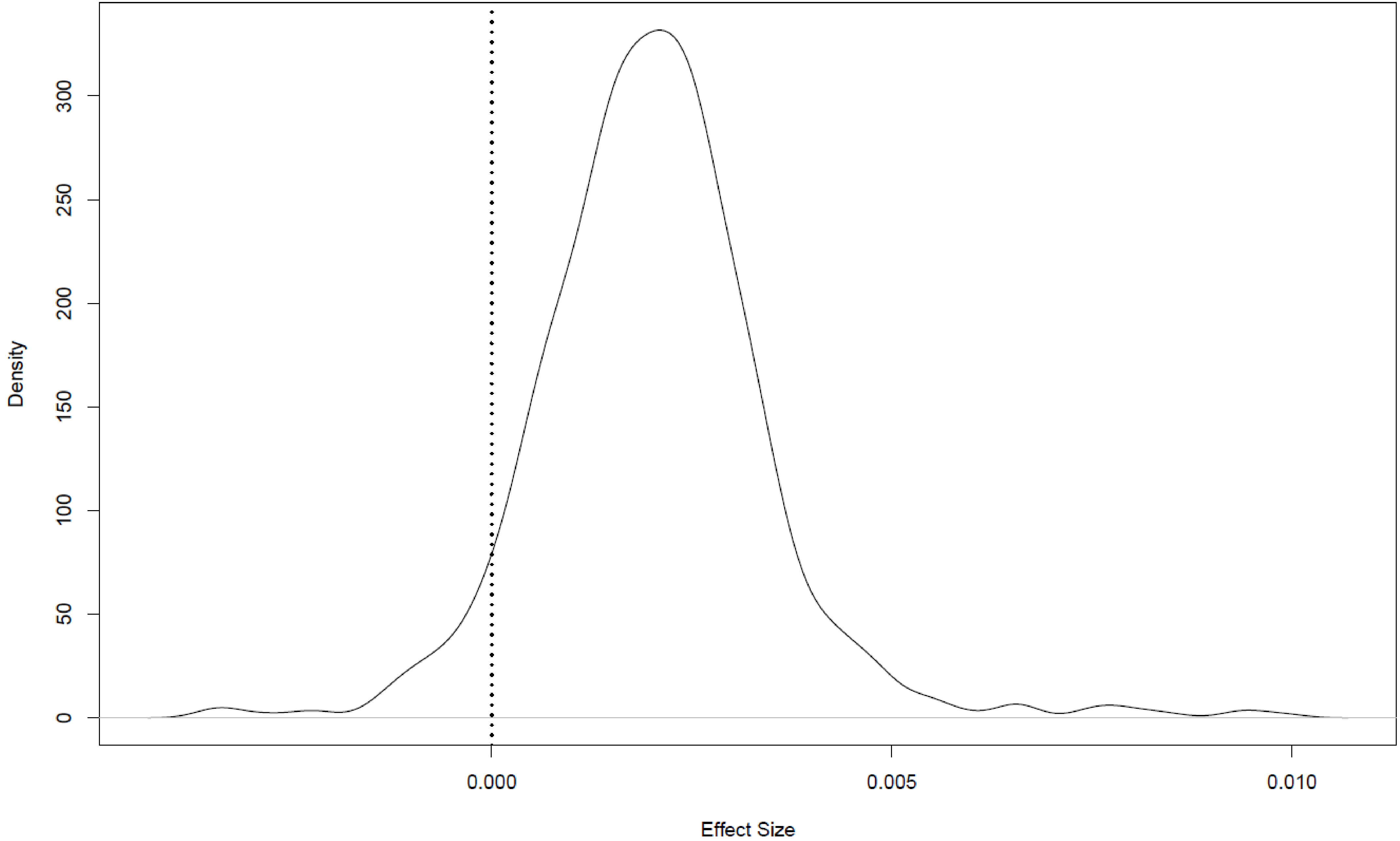

Table S1. Sampling year and location of the isolates collected along the Niger River.

Isolate*	Year	Location				
		Country	Region	Rice perimeter	Latitude	Longitude
Ng4	1997	Niger	Tillabéri	Bonféba	14.39	1.19
Ng11	2003	Niger	Tillabéri	Namari	14.35	1.25
Ng8	2003	Niger	Tillabéri	Toula	14.19	1.46
Ng111	2007	Niger	Tillabéri	Daïkaina	14.18	1.48
Ng250	2008	Niger	Tillabéri	Daïkaina	14.18	1.48
Ng109	2007	Niger	Tillabéri	Daïberi	14.16	1.50
Ng1101	2008	Niger	Tillabéri	Daïberi	14.16	1.50
Ng335	2007	Niger	Tillabéri	Kokomani	14.00	1.52
Ng101	2007	Niger	Niamey	Koutoukalé	13.69	1.73
Ng321	2007	Niger	Niamey	Koutoukalé	13.69	1.73
Ng7	2003	Niger	Niamey	Karma	13.66	1.84
Ng9	2003	Niger	Niamey	Karaïgourou	13.58	1.96
Ng105	2007	Niger	Niamey	Karaïgourou	13.58	1.96
Ng115	2007	Niger	Niamey	Karaïgourou	13.58	1.96
Ng18	2005	Niger	Niamey	Goudel	13.53	2.03
Ng20	2005	Niger	Niamey	Goudel	13.53	2.03
Ng21	2005	Niger	Niamey	Goudel	13.53	2.03
Ng22	2005	Niger	Niamey	Goudel	13.53	2.03
Ng23	2005	Niger	Niamey	Goudel	13.53	2.03
Ng24	2005	Niger	Niamey	Goudel	13.53	2.03
Ng25	2005	Niger	Niamey	Goudel	13.53	2.03
Ng27	2005	Niger	Niamey	Goudel	13.53	2.03
Ng28	2005	Niger	Niamey	Goudel	13.53	2.03
Ng29	2005	Niger	Niamey	Goudel	13.53	2.03
Ng30	2005	Niger	Niamey	Goudel	13.53	2.03
Ng3	1998	Niger	Niamey	Kirkissoye	13.49	2.11
Ng106	2007	Niger	Niamey	Saga	13.46	2.12
Ng10	2003	Niger	Niamey	Libore	13.40	2.19
Ng103	2007	Niger	Niamey	N'Dounga	13.37	2.22
Ng215	2008	Niger	Niamey	N'Dounga	13.37	2.22
Ng119	2007	Niger	Niamey	Kollo	13.34	2.30
Ng110	2007	Niger	Niamey	Seberi	13.29	2.34
Ng104	2007	Niger	Niamey	Say-1	13.10	2.35
Ng114	2007	Niger	Niamey	Say-1	13.10	2.35
Ng201	2008	Niger	Niamey	Say-1	13.10	2.35
Ng6	1998	Niger	Niamey	Say-2	13.10	2.35
Ng113	2007	Niger	Gaya	Tara	11.89	3.33
Be1	1999	Benin		Malanville	11.87	3.38
Be2	1999	Benin		Malanville	11.87	3.38
Be3	2003	Benin		Malanville	11.87	3.38
Be4	2005	Benin		Malanville	11.87	3.38
Be5	2005	Benin		Malanville	11.87	3.38
Be6	2005	Benin		Malanville	11.87	3.38
Be27	2006	Benin		Malanville	11.87	3.38

* Niger (Ng), Benin (Be).

Table S2. Accession numbers of the coat protein gene and full-length sequences of the isolates of the WA261 dataset.

Isolate[1]	Accession number	Isolate	Accession number	Isolate	Accession number	Isolate	Accession number	Isolate	Accession number	Isolate	Accession number	Isolate	Accession number
Be1	AJ885087	Ce17	MF066664	CI15	AJ279916	Gu1	AJ279920	Ma185	AJ885145	Ng30*	MZ172930	SL3	AJ279937
Be2	AJ885088	Ce19	MF066665	CI16	AJ279917	Gu21	AJ885124	Ma201	AM931178	Ng101*	MZ172963#	SL4	AJ608214#
Be3	AM931172	Ce20	MF066666	CI17	AJ279918	Gu26	AJ885125	Ma202	AM931180	Ng102*	MZ172960#	SL5	AJ885152
Be4	FN432842	Ce22	MF066667	CI46	AJ885094	Gu28	AJ885126	Ma203	FN432840#	Ng103*	MZ172931	SL6	AJ279934
Be5	FN432843	Ce26*	MZ172955#	CI47	AJ885095	Gu29	AJ885127	Ma204	AM931182	Ng104*	MZ172932	SL7	AJ885153
Be6	FN432844	Ce28	MF066670	CI63	AJ608207#	Gu30	AM931176	Ma205	AM931183	Ng105	MZ172964#	Tc11	AJ317952
Be27	FN432845	Ce31	KF054745	CI65	AJ885096	Gu31	AM931177	Ma206	AM931184	Ng106*	MF784437#	Tc17	AJ317953
Be120*	MZ172951	Ce32	KF054742	CI66	AJ885097	Gu32	AM931178	Ma207	AM931185	Ng109	MF784438	Tc24	AJ317954
Be123*	MZ172952	Ce37	MF066675	CI67	AJ885098	Gu101	FN432854	Ma208	AM931186	Ng110*	MZ172933	Tc28	FN432837#
Be125*	MZ172961#	Ce46	MF066676	CI68	AM931175	Gu107	FN432855	Ma209	AM931187	Ng111*	MZ172934	Tc31	FN432850
Be127*	MZ172956#	Ce61	MF066681	CI101	AJ885099	Gu111	FN432856	Ma210	AJ885146/	Ng113*	MF461282	Tc32	FN432851
Be136*	MZ172957#	Ce63	MF066682	CI104	AJ885100	Gu201	FN432857	Ma274*	MZ172921	Ng114*	MZ172935	Tc33	FN432852
BF1	AM883059#	Ce64	MF066683	CI105	AJ885101	Ma1	AJ279926	Ma301	FN432860	Ng115*	MZ172936	Tc34	FN432853
BF2	AJ885089	Ce66*	MZ172953	CI106	AJ885102	Ma2	AJ279927	Ma302	FN432861	Ng119*	MZ172965#	Tg1*	MZ172966#
BF5*	MZ172956#	Ce72	MF066684	CI109	AJ885103	Ma3	AJ279928	Ma303	FN432862	Ng201*	MZ172940	Tg4	AJ885167
BF570	AJ885091	Ce93	MF066689	CI110	AJ885104	Ma4	AJ279929	Ma304	FN432863	Ng215*	MZ172941	Tg7	AJ885168
BF572	AJ885092	Ce163	MF066693	CI111	AJ885105	Ma5	AJ279930	Ma305	FN432864	Ng217*	MZ172943	Tg9	AJ885169
BF682	AJ885093	Ce170	MF066694	CI112	AJ885106	Ma6	AJ279931	Ma389*	MZ172923	Ng218*	MZ172954#	Tg12	AJ885170
BF702	AM931174	Ce175	MF066695	CI113	AJ885107	Ma7	AJ885129	Ma403*	MZ172924	Ng221*	MZ172944	Tg21	AJ885172
BF801*	MZ172959#	Ce177	MF066697	CI114	AJ885108	Ma8	AJ885130	Ma421*	MZ172925	Ng250*	MZ172942	Tg247	MF784439
BF802	FN432847	Ce181	MF066698	CI115	AJ885109	Ma9	AJ885131	Ma479*	MZ172928	Ng321*	MZ172937	Tg274	MF784441#
BF803	FN432848	Ce183	MF066699	CI116	AJ885110	Ma10	AJ608208#	Ma701*	MZ172970#	Ng335*	MZ172938		
BF804	FN432849	Ce226	MF066669	CI117	AJ885111	Ma25*	MZ172920	Ng3	AJ885147	Ng1101*	MZ172939		
BF810*	MZ172958#	Ce228	MF066671	CI118	AJ885112	Ma29	AJ885132	Ng4	AJ885148	Nia	U23142#		
Ca2*	MZ172917	CIa	AJ608219#	CI121	AJ885113	Ma34*	MZ172922	Ng6	AJ885149	Ni1	AJ608212#		
Ca5*	MZ172967#	CIb	L20893#	CI129	AJ885114	Ma41	AJ885133	Ng7	AM931215	Ni2	AJ608213#		
Ca12*	MZ172914	CI1	AJ279902	CI138	AJ885115	Ma43*	MZ172926	Ng8	AM931216	Ni4	AJ885150		
Ca21*	MZ172915	CI2	AJ279903	CI139	AJ885116	Ma45*	MZ172927	Ng9	AM931217	Ni6	AJ885151		
Ca22*	MZ172916	CI3	AJ279904	CI151	AJ885117	Ma77	AJ608209#	Ng10	AM931218	Ni106*	MZ172945		
Ca30	AJ317950	CI4	AJ608206#	CI152	AJ885118	Ma98	AJ885134	Ng11	AM931219	Ni107*	MZ172946		
Ca38	AJ306735	CI5	AJ279906	CI153	AJ885119	Ma105*	MZ172969#	Ng18	FN432841#	Ni108*	MZ172947		
Ca43*	MZ172918	CI6	AJ279907	CI154	AJ885120	Ma144	AJ885136	Ng20	FN432865	Ni111*	MZ172948		
Ca52*	MZ172919	CI7	AJ279908	CI155	AJ885121	Ma145	AJ885137	Ng21	FN432866	Ni118*	MZ172949		
Ca54	AJ317951	CI8	AJ279909	CI156	AJ885122	Ma146	AJ885138	Ng22	FN432867	Ni124*	MZ172950		
Ce1	KF054740	CI9	AJ279910	CI157	AJ885123	Ma148	AJ885139	Ng23	FN432868	Ni131	AJ884691		
Ce2	KF054741	CI10	AJ279911	Ga1	AM765810	Ma149	AJ885140	Ng24	FN432869	Se1	MN233654#		
Ce4	KF054743	CI11	AJ279912	Ga2	AM765811	Ma171	AJ885141	Ng25*	MZ172929	Se5	MN233655#		
Ce5	MF066660	CI12	AJ279913	Ga3	AM765812	Ma175	AJ885142	Ng27	FN432870	Se8	MH699983		
Ce13	MF066661	CI13	AJ279914	Ga4	FN432838#	Ma179	AJ885143	Ng28	FN432871	SL1	AJ279935		
Ce14*	MZ172968#	CI14	AJ279915	Gh1	AJ279919	Ma180	AJ885144	Ng29	FN432872	SL2	AJ279936		

[1] Benin (Be), Burkina-Faso (BF), Cameroon (Ca), Central African Republic (Ce), Mali (Ma), Republic of Niger (Ng), Nigeria (Ni), Togo (Tg).

* from this study.

# full-length sequences.

Table S3. Accession numbers of the coat protein gene sequences of the isolates of the EA240 dataset.

Isolate[1]	Accession number	Isolate	Accession number	Isolate	Accession number	Isolate	Accession number	Isolate	Accession number	Isolate	Accession number
Bu1	HE654712	Rw104	HQ650138	Tz122	AJ884688	Tz407	MF447488	Tz801	MF447529	Tz2085	KM487714
Bu2	HE654713	Rw105	HQ650139	Tz124	AJ884702	Tz408	MF447489	Tz820	AB740032	Tz3026	KM487715
Bu4	HE654714	Rw110	HE654711	Tz125	AJ884701	Tz416	MF447490	Tz836	AB740031	Ug1	AM114523
Bu7	HE654715	Rw181	HE654703	Tz127	AJ876793	Tz421	MF447491	Tz1004	FN432880	Ug2	AM114524
Bu10	HE654716	Rw201	HE654721	Tz128	AJ884689	Tz429	MF447492	Tz1017	FN432881	Ug18	KM487716
Bu13	HE654718	Rw205	HE654722	Tz129	AJ884713	Tz441	MF447493	Tz1032	FN432882	Ug50	KM487717
Bu16	HE654719	Rw206	HE654723	Tz130	AJ884699	Tz445	MF447494	Tz1041	FN432883	Ug101	KM487718
Bu17	HE654717	Rw208	HE654720	Tz200	AM931220	Tz449	MF447495	Tz1044	HE963829	Ug102	KM487719
Co209	KC788208	Tz1	AJ279938	Tz201	AM931221	Tz450	MF447496	Tz1048	FN432884	Ug103	KM487720
Co223	KC788209	Tz2	AJ279939	Tz202	AM883057	Tz452	MF447497	Tz1051	FN432885	Ug104	KM487721
Co229	KC788210	Tz3	AJ279940	Tz203	AM931222	Tz454	MF447498	Tz1061	FN432886	Ug105	KM487738
Et2	KM017554	Tz4	AJ511793	Tz204	AM931223	Tz460	MF447499	Tz1066	HE963828	Ug107	KM487722
Et3	KM017555	Tz5	AJ511794	Tz205	AM931224	Tz461	MF447500	Tz1067	HE963830	Ug108	KM487723
Et5	KM017556	Tz6	AJ511795	Tz206	AM931225	Tz463	MF447501	Tz1069	FN432887	Ug109	KM487724
Et15	MH917947	Tz7	AJ511796	Tz207	AM883097	Tz483	MF447502	Tz1070	FN432888	Ug110	KM487725
Et19	MH917949	Tz8	AJ511797	Tz208	AM931227	Tz486	MF447503	Tz1072	FN432889	Ug142	KM487726
Et20	MH917950	Tz9	AJ511798	Tz209	AM883058	Tz503	MF447504	Tz1073	FN432890	Ug143	KM487727
Et21	KM017557	Tz10	AJ511799	Tz210	AM931228	Tz504	MF447505	Tz1074	FN432891	Ug144	KM487728
Ke1	AJ511805	Tz11	AJ511800	Tz211	AM931229	Tz507	MF447506	Tz1075	FN432892	Ug145	KM487729
Ke2	AJ885128	Tz12	AJ511801	Tz212	AM931230	Tz508	MF447507	Tz1077	FN432893	Ug146	KM487730
Ke3	AM931178	Tz13	AJ885154	Tz213	AM931231	Tz510	MF447508	Tz1082	FN432894	Ug148	KM487711
Ke11	FN432857	Tz14	AJ885155	Tz214	AM931232	Tz512	MF447509	Tz1086	FN432895	Ug201	KM487731
Ke12	FN432858	Tz15	AJ885156	Tz215	AM931233	Tz515	MF447510	Tz1094	FN432896	Ug204	KM487732
Ke13	FN432859	Tz16	AJ885157	Tz216	AM931234	Tz516	MF447511	Tz1095	HE963827	Ug205	KM487733
Ke323	MG599279	Tz17	AJ885158	Tz217	AM931235	Tz520	MF447512	Tz1099	FN432897	Ug207	KM487712
Ke345	MG599280	Tz18	AJ885159	Tz218	AM931236	Tz523	MF447513	Tz1101	FN432898	Ug210	KM487734
Mw2	KP274893	Tz19	AJ885160	Tz219	AM931237	Tz525	MF447514	Tz1102	FN432899	Ug211	KM487735
Mw3	KP274894	Tz20	AJ885161	Tz220	AM931238	Tz526	MF447515	Tz1103	FN432900	Ug212	KM487736
Mw4	KP274895	Tz21	AJ885162	Tz221	AM931239	Tz533	MF447516	Tz1107	FN432901	Ug220	KM487737
Mw5	KP274896	Tz22	AJ885163	Tz222	AM931240	Tz539	MF447517	Tz1110	FN432902	Ug230	KM487713
Mw10	MF989228	Tz24	AJ885165	Tz223	AM931241	Tz543	MF447518	Tz1112	FN432903	Ug401	AB981479
Mw13	KP274897	Tz101	AJ884693	Tz225	FN432873	Tz554	MF447519	Tz1113	FN432904	Ug402	AB985686
Rw1	GQ470541	Tz102	AJ884705	Tz226	FN432874	Tz601	MF447520	Tz1114	FN432905	Ug408	AB980006
Rw2	GQ470542	Tz104	AJ884690	Tz229	FN432877	Tz607	MF447521	Tz1116	FN432906	Ug410	AB980004
Rw15	HE654704	Tz109	AJ884694	Tz231	FN432878	Tz612	MF447523	Tz1118	FN432907	Ug412	AB980003
Rw19	HE654706	Tz111	AJ884714	Tz232	FN432879	Tz615	MF447524	Tz1120	FN432908	Ug413	AB980005
Rw30	HE654709	Tz112	AJ884712	Tz401	MF447484	Tz619	MF447525	Tz1122	FN432909	Ug414	AB980010
Rw101	HQ650135	Tz113	AJ884711	Tz402	MF447485	Tz651	MF447526	Tz1125	FN432910	Ug421	AB980007
Rw102	HQ650136	Tz118	AJ884707	Tz403	MF447486	Tz701	MF447527	Tz1127	FN432911	Ug425	AB980008
Rw103	HQ650137	Tz121	AJ884704	Tz405	MF447487	Tz702	MF447528	Tz1130	FN432912	Ug439	AB980009

[1] Burundi (Bu), Republic of Congo (Co), Ethiopia (Et), Kenya (Ke), Malawi (Mw), Tanzania (Tz), Uganda (Ug).

Table S4. Sequence accession numbers of the coat protein gene of the isolates of the Mg94 dataset.

Isolate[1]	Accession number	Isolate	Accession number	Isolate	Accession number
Mg1	AJ279921	Mg48	JX961559	Mg210	JX961598
Mg2	AJ279922	Mg49	JX961560	Mg301	MK942094
Mg3	AJ279923	Mg50	JX961561	Mg302	MK942095
Mg4	AJ279924	Mg51	JX961562	Mg303	MK942096
Mg5	AJ279925	Mg52	JX961563	Mg304	MK942097
Mg6	AM931189	Mg53	JX961564	Mg341	MK942098
Mg10	AM931190	Mg54	JX961565	Mg342	MK942099
Mg11	AM931191	Mg55	JX961566	Mg343	MK942100
Mg12	AM931192	Mg56	JX961567	Mg344	MK942101
Mg13	AM931192	Mg57	JX961568	Mg401	MK942104
Mg14	AM931194	Mg58	JX961569	Mg402	MK942102
Mg15	AM931195	Mg59	JX961570	Mg403	MK942103
Mg16	AM883056	Mg60	JX961571	Mg410	MK942106
Mg17	AM931196	Mg61	JX961572	Mg419	MK942105
Mg18	AM931197	Mg62	JX961573		
Mg19	AM931198	Mg63	JX961574		
Mg20	AM931199	Mg64	JX961575		
Mg21	AM931200	Mg65	JX961576		
Mg22	AM931201	Mg66	JX961577		
Mg23	AM931202	Mg67	JX961578		
Mg24	AM931203	Mg68	JX961579		
Mg25	AM931204	Mg69	JX961580		
Mg26	AM931205	Mg70	JX961581		
Mg27	AM931206	Mg71	JX961582		
Mg28	AM931207	Mg105	JX961583		
Mg29	AM931208	Mg108	MK942093		
Mg30	AM931209	Mg119	JX961584		
Mg31	AM93210	Mg123	JX961585		
Mg32	AM93211	Mg124	JX961586		
Mg33	AM93212	Mg125	JX961587		
Mg34	AM93213	Mg126	JX961588		
Mg35	AM93214	Mg127	JX961589		
Mg40	JX912946	Mg128	JX961590		
Mg41	JX961552	Mg147	JX961591		
Mg42	JX961553	Mg150	JX961592		
Mg43	JX961554	Mg155	JX961593		
Mg44	JX961555	Mg163	JX961594		
Mg45	JX961556	Mg165	JX961595		
Mg46	JX961557	Mg201	JX961596		
Mg47	JX961558	Mg202	JX961597		

[1] Madagascar (Mg).
